# Vimentin and Anti Vimentin Antibodies in Chagas' Disease

**DOI:** 10.5935/abc.20180038

**Published:** 2018-04

**Authors:** Marilda Savoia Nascimento, Anna Maria Simonsen Stolf, Heitor Franco de Andrade Junior, Ramendra Pati Pandey, Eufrosina Setsu Umezawa

**Affiliations:** Universidade de São Paulo (USP), São Paulo, SP - Brazil

**Keywords:** Chagas Disease, Heart Diseases, Trypanossoma Cruzi, Rheumatic Fever, Vimentin, Antibodies, Monoclonal

## Abstract

**Background:**

Vimentin is a main structural protein of the cell, a component of intermediate cell filaments and immersed in cytoplasm. Vimentin is mimicked by some bacterial proteins and anti-vimentin antibodies occur in autoimmune cardiac disease, as rheumatic fever. In this work we studied vimentin distribution on LLC-MK2 cells infected with T. cruzi and anti-vimentin antibodies in sera from several clinical pictures of Chagas' disease or American Trypanosomiasis, in order to elucidate any vimentin involvement in the humoral response of this pathology.

**Objective:**

We standardized an indirect immunofluorescence assay (IFI) to determine sub cellular expression in either parasites and host cells, and ELISA to evaluate anti-vimentin antibodies in sera fron chagasic patients.

**Methods:**

We analyzed the distribution of vimentin in culture cells using indirect fluorescent assays, using as external controls anti-T. cruzi sera, derived from chronic infected patients for identification of the parasites in the same model. After infection and growth of T.cruzi amastigotes, those cells express larger amounts of vimentin, with heavy staining of cytoplasm outside the parasitophorous vacuole and some particle shadowing patterns, suggesting that vimentin are associated with cell cytoplasm. Anti-vimentin antibodies were present in most American trypanosomiasis samples, but notably, they are much more present in acute (76, 9%) or clinical defined syndromes, especially cardiac disease (87, 9%). Paradoxically, they were relatively infrequent in asymptomatic (25%) infected patients, which had a clearly positive serological reaction to parasite antigens, but had low frequency of anti-vimentin antibodies, similar to controls (2,5%).

**Conclusion:**

Our current data revealed that anti-vimentin antibodies induced during T. cruzi infection could be a marker of active disease in the host and its levels could also justify drug therapy in American Trypanosomiasis chronic infection, as a large group of asymptomatic patients would be submitted to treatment with frequent adverse reactions of the available drugs. Anti-vimentin antibodies could be a marker of cardiac muscle cell damage, appearing in American Trypanosomiasis patients during active muscle cell damage.

## Introduction

Chagas' disease or American Trypanosomiasis is a peculiar parasitic infection as *Trypanosoma cruzi* is a unique intracellular parasite which resulted in cytoplasmic presence of amastigotes forms, a rare cellular event in nature, as cytoplasm is usually free from parasites in almost all intracellular infections^1^. After its reproduction, the parasite had a set of enzymes, as sialidases, that transfers host cell molecules to their surface, allowing cell evasion without disruption^2^. All those processes could alter cell cytoskeleton and its proteins, probably generating in the host cell signals that alters the protein synthesis of structural proteins. Vimentin is a main structural protein of the cell, a component of intermediate cell filaments and immersed in cytoplasm^3^. Vimentin is expressed in normal cardiac muscle and their tumors, and autoantibodies against a vimentin re found in allograft rejection^4^. or cardiac models of allograft rejection^5^.Vimentin is mimicked by some bacterial proteins and anti-vimentin antibodies occur in autoimmune cardiac disease, as rheumatic fever^6^. In this work we studied vimentin distribution on LLC-MK2 cells infected with *T.cruzi* and anti-vimentin antibodies in sera from several clinical pictures of American Trypanosomiasis, in order to elucidate any vimentin involvement in the humoral response of this pathology.

## METHODS

### Parasites and serum samples

*Trypanosoma cruzi* epimastigotes were grown from Y strain routinely maintained in our lab on Liver Infusion Tryptose (LIT) culture media supplemented with 10% fetal calf serum. *T. cruzi* trypomastigotes were obtained from cell culture supernatants of LLC-MK2 cells previously infected. Monoclonal mouse Anti-Vimentin antibody (V6630) and vimentin from bovine lens was obtained commercially (Sigma Aldrich, Saint Louis, Missouri, USA). A serum from patient with cardiac chronic American Trypanosomiasis was used as anti *T.cruzi* antibody. Human sera from American Trypanosomiasis patients and controls were used from the biorepository of *T.cruzi* patients samples from E.S.Umezawa, Lab.Protozoology, IMTSP, serologically characterized in TESA specific serology tests and published previously in several articles, were recovered and comprising 26 sera from acute disease, 33 from isolated cardiac disease, 17 from isolated digestive disease, 20 without clinical disease (asymptomatic disease) and 40 sera from patients outside endemic area. All clinical data were maintained by the attendant physician and not available for this study.

### Antigen expression and morphology

All morphological assays were performed in a Zeiss Axioplan epifluorescent microscope with fluorescein filters. For antigen detection, we fixed LLC-MK2 control cells, *T.cruzi* infected LLC-MK2 cells and *T.cruzi* epimastigotes and permeated cell surface with Triton X-100^7^ with either anti-Vimentin mAb or anti-*T.cruzi* antibodies as elsewhere described. After this step, bound antibodies were revealed with adequate fluorescein conjugate, carefully washed and mounted in glycerin for observation. Representative Fields were digitalized at high power field using a Canon camera. 

### TESA and vimentin ELISA

*T.cruzi* trypomastigotes excreted secreted antigen was obtained as elsewhere described^8^. TESA (1/80) and Vimentin (0.06ug/ml) in carbonate 0.05 M pH9.6 were adsorbed overnight to wells of 96 wells high binding ELISA plates (Corning Inc. New York, USA). After washing and blocking with PBS Tween 20, 0,05% plus 5% milk or BSA 0.5%, adequate dilution of sera (1/50 vimentin and 1/200 TESA) were incubated for one hour. After new washings, adequate dilution of peroxidase conjugate were added for another hour, washed and bound conjugate revealed by 1 h with orto-phenylenediamine and hydrogen peroxide. After 30 min in 37ºC, reaction was stopped with 4N HCl and 492 nm absorbance determined in a microplate reader (Multiskan-Titertek II).

### Statistical analysis

All quantitative data, such as O.D. ELISA, were analyzed using ANOVA after the Levene test for variance check, with intragroup comparisons by the Bonferroni's test, if there are uniformity of variances. In the absence of this homogeneity, data were analyzed by Kruskal-Wallis tests with Dunns post-tests. We opt for graphical representation of individual data in dot plot with association of mean and SEM for comparison. Qualitative analysis, as frequency of positive sera in the group, was analyzed by Fisher exact tests in two group analysis. We also included 95% confidence interval of estimated proportion. Significant difference was considered when the probability of equality (H1 = H0) was less than 0.05(p≤0.05), using two-tailed analysis and power greater than 90%. We used the statistical package GraphPad Prism 7.0 for all statistical analysis and plotting.

## Results

**Figure 1:**
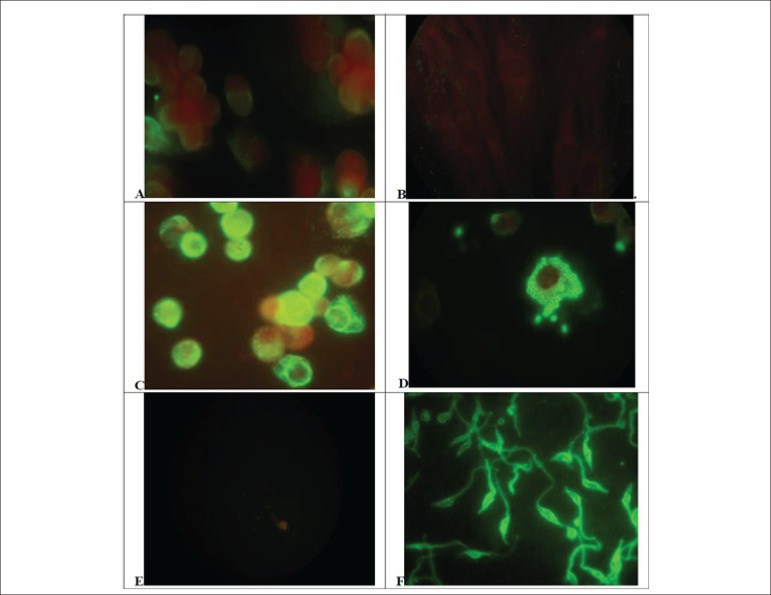
Distribution of Vimentin or Trypanosoma cruzi antigen on control or infected cells and parasites. A and B) Uninfected LLC MK2 cells reacted to antivimentin abs(A) or anti-T.cruzi abs(B). C and D) T.cruzi infected LLC MK2 cells reacted to antivimentin abs(C) or anti-T.cruzi abs(D). E and F) T.cruzi promastigote forms from in vitro culture reacted to antivimentin abs (E) or anti-T.cruzi abs(F). Cells, infected cells or parasites forms were reacted with Anti Vimentin mAb or chronic infection chagasic serum and revealed with adequate conjugate (x1000) (see Methods).

We analyzed the distribution of Vimentin in culture cells using indirect fluorescent assays as described in Methods, using as external controls anti-*T.cruzi* sera, derived from chronic infected patients for identification of the parasites in the same model, as could be seen in figure 1. LLC-MK2 cells, the host cell used for intracellular growth of *T.cruzi*, showed a discrete and uniform cytoplasmic staining, uniform in most cells (Figure 1A). Those cells are no reactive to anti-*T-cruzi* antibodies, without any staining (Figure 1B). After infection and growth of *T.cruzi* amastigotes, those cells express larger amounts of vimentin, with heavy staining of cytoplasm outside the parasitophorous vacuole and some particle shadowing patterns, suggesting that vimentin are associated with cell cytoplasm (Figure 1C). Vimentin could involve unstained cytoplasmic parasites, but no specific staining of parasites was seen. Those parasites were easily identified by anti-*T.cruzi* antibodies showing a typical morular pattern in the cytoplasm of infected cells (Figure 1D). No staining of those parasites was observed with anti-vimentin mAbs, which demonstrate the absence of antigen mimicry, both for amastigotes (Figure 1C) or extracellular parasites (Figure 1E). Those extracellular parasites are heavily stained by anti *T.cruzi* antibodies as well as intracellular amastigotes (Figure 1F). 

### Anti-vimentin auto antibodies

**Figure 2:**
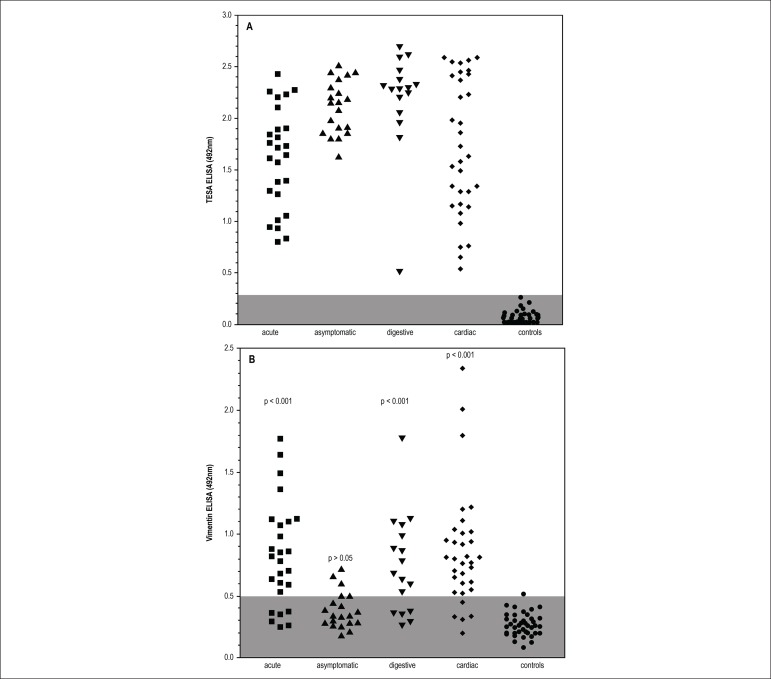
Sera reactivity profile of patients with different clinical forms of Chagas disease by ELISA with T. cruzi TESA antigens (A) and commercial vimentin (B). Groups were compare with ANOVA with Bonferronipost tests.

We search for anti-vimentin antibodies in human sera from controls or American trypanosomiasis patients. Our sample was composed by patients with well-defined clinical forms as described in Methods. Vimentin ELISA was prepared with commercial protein and antibody binding was revealed by commercial conjugates. Standardization was easily as controls were adequately negative, allowing an adequate cut-off definition. We also tested all samples in a reported high specificity ELISA assay, TESA, which uses an excreted secreted antigen in solid phase, with high reactivity in all clinical forms of American trypanosomiasis. Those assays could be seen in Figure 2. We clearly demonstrate that all patients from our sample of American trypanosomiasis react very well in TESA assay, without any false positive or dubious sample in control groups. All clinical forms presented a similar reactivity for parasite antigens, including those with asymptomatic infection. Anti-vimentin antibodies were present in most American trypanosomiasis samples, but notably, they are much more present in acute or clinical defined syndromes (76,9%), especially cardiac disease (87,9%) and digestive form 70,5%. Paradoxically, they were relatively infrequent in asymptomatic infected patients (25%), which had a clearly positive serological reaction to parasite antigens, but had low frequency of anti-vimentin antibodies, similar to controls (2,5%), p>0.05, Bonferroni's ANOVA post-test) or frequency of positive samples (Table 1) with similar conclusions. The main reactivity of these autoantibodies appears to be more intense in active cardiac or acute disease, which were associated to large parasite burden and inflammatory response than digestive or asymptomatic disease. The proportion of reagent sera was also shown in Table 1 assuming cutoff value estimated as defined in methods. The anti-vimentin ELISA reactivity of sera from patients with clinical active disease for any origin was in higher frequency than in patients without active disease or non-infected controls. Data were compared mainly with active or undetermined without clinical forms of Chagas' disease shows greater difference as expected with high statistical difference (p < 0.01) and also demonstrated by 95% confidence interval of the proportion

**Table 1:** Percentage of positivity of sera with different clinical forms of Chagas disease for Vimentin antigen in the ELISA reaction

Clinical form	Samples (n)	Positives (n)	Negatives (n)	Positivity (%)	95% C.I. (p vs w/o Chagas)	p VS undetermined
Acute	26	20	6	76.9	53-87 (p < 0.001)	p < 0.001
Cardiac	33	29	4	87.8	67-93 (p < 0.001)	p < 0.001
Digestive	17	12	5	25	42-84 (p < 0.001)	p < 0.01
Symptomatic	76	61	15	80,2	70-88 (p < 0.001)	p < 0.001
Undetermined	20	5	15	70,5	5-44 (p < 0.05)	
Total Chagas	96	66	30	68,5	52-75 (p < 0.001)	
Without Chagas	40	1	39	2,5	1-4%	

*Fisher exact test

The Table 1 Summarizes the data obtained in Figure 2 and provides ELISA positivity indexes with commercial Vimentin, showing that the percentage of positive sera from the groups of chronic patients with clinical manifestations of Chagas' disease and the group of patients from the acute phase was higher than that observed in the indeterminate group of chagasic patients.

The positivity index of sera from patients in the acute phase was 76.9% with 20 positive sera from the 26 analyzed. In the group of chronic digestive tract positive percentage was 70.5% with 12 positive in the 17 analyzed, the cardiac patients had a positive percentage of 87.9% with 29 positive sera from the 23 analyzed and in the group of indeterminate patients, the Index was 25% with 5 positive of the 20 analyzed. The positivity of the non-chagasic sera was 2.5% or only a positive serum in 40 analyzed.

## Discussion

This intracytoplasmic infection resulted in altered expression of cell fibrillary proteins as vimentin, as we clearly show in immunofluorescence of infected cells. This altered vimentin production is devoid of association with the parasite, which has no reactivity with antivimentin antibodies in any form. Vimentin is important for specific virus entry, another possible cytoplasmic pathogen and are used by Foot-and-mouth disease virus (FMDV) for virus mounting inside the cells.^9^

This intracytoplasmic infection resulted in altered expression of cell fibrillary proteins as vimentin, as we clearly show in immunofluorescence of infected cells. This altered vimentin production is devoid of association with the parasite, which has no reactivity with anti-vimentin antibodies in any form. Viral infection alters host cell architecture similarly, as parvovirus in mice^10^ but other pathogens also affects vimentin distribution in infected cells, with similar perivacuolar distribution, as in Salmonella infections.^11^ Proteomics studies in experimental models of *T.cruzi* infection had shown higher plasma levels of vimentin related to disease severity,^12^ which can offer to the immune response intracellular filaments for antibody production. Those data were expected as vimentin autoantibodies could be related to antigen exposure during active infection, as proposed in experimental models of *T.cruzi* infection.^12^ Several other immune diseases that interact with cardiac muscle cells also presented anti-vimentin antibodies. Murine models of viral myocarditis presented those antibodies^13^ and as well as post-streptococcal rheumatic fever patients^14^. Noninfectious myocarditis, as in coronary artery disease patients^15^ and kidney or heart transplants recipients^16^ also showed those antibodies resulted from any exposure of antigen, unregard the origin of cardiac muscle cell damage. Our data were similar to those findings and anti-vimentin antibodies induced during *T.cruzi* infection could be a marker of active disease in the host and its levels could also justify drug therapy in American Trypanosomiasis chronic infection, as a large group of asymptomatic or indeterminate patients would be submitted to treatment with frequent adverse reactions of the available drugs. Anti-vimentin antibodies could be a marker of cardiac muscle cell damage, appearing in American Trypanosomiasis patients during active muscle cell damage.

## Conclusions

Our data revealed that anti-vimentin antibodies induced during activity of *T. cruzi* infection could be a marker of active disease in the host, despite absence of evident clinical involvement. This assay could be also a non-invasive follow-up test during drug therapy in Chagas' disease or American Trypanosomiasis. This test could allow the selection of possible active patients for therapy and also to supply a marker of disease activity after therapy, avoiding that a large group of asymptomatic patients without active disease were submitted to treatment with frequent adverse reactions. Anti-vimentin antibodies could be a marker of cardiac muscle cell inflammatory involvement, showed by American Trypanosomiasis patients with active muscle cell damage and must be tested in other cardiac muscle inflammatory conditions as viral myocarditis.
